# A Rare Case of Pylephlebitis as a Complication of Cholecystocolonic Fistula

**DOI:** 10.1155/2018/3931674

**Published:** 2018-11-04

**Authors:** Kouki Imaoka, Saburo Fukuda, Hirofumi Tazawa, Sotaro Fukuhara, Yuzo Hirata, Seiji Fujisaki, Mamoru Takahashi, Hideto Sakimoto

**Affiliations:** ^1^Department of Surgery, Chugoku Rosai Hospital, 1-5-1 Hirotagaya, Kure, Hiroshima 737-0193, Japan; ^2^Department of Surgery, Hiroshima City Citizens Hospital, 7-33 Motomachi, Naka-ku, Hiroshima 730-8518, Japan; ^3^Department of Surgery, National Hospital Organization Kure Medical Center and Chugoku Cancer Center, 3-1 Aoyama, Kure, Hiroshima 7007-0023, Japan; ^4^Faculty of Medicine, Hiroshima University, 1-2-3 Kasumi, Hiroshima 734-8551, Japan

## Abstract

Pylephlebitis is defined as a septic thrombophlebitis of the portal vein and its tributaries that is associated with multiple suppurative abdominal infections. We report a case of pylephlebitis associated with a cholecystocolonic fistula (CCF). A 41-year-old man presented with upper abdominal pain and anorexia for 1 month. Abdominal contrast-enhanced computed tomography (CT) revealed thrombosis in the left and anterior branch of the portal vein and thickening of the walls of the portal vein and periside portals. The gallbladder was collapsed and pneumobilia was seen in the biliary tract. Blood culture was positive for *Streptococcus anginosus*. A diagnosis of thrombophlebitis of the portal vein associated with CCF was made, and the patient was immediately managed with an intravenous broad-spectrum antibiotic and anticoagulation. After the portal vein thrombosis (PVT) propagation and inflammation had subsided, cholecystectomy and partial resection of the transverse colon were performed. Pylephlebitis is rare but is a life-threatening complication of intra-abdominal infection. A high index of suspicion is required, and a CT scan should be performed immediately for an early diagnosis and appropriate treatment.

## 1. Introduction

Pylephlebitis is defined as a septic thrombophlebitis of the portal vein and its tributaries that is associated with intra-abdominal inflammation and infections such as diverticulitis, appendicitis, cholangitis, and cholecystitis [1]. Although pylephlebitis is a rare disease, the mortality rate has been reported to be 30%–50% [2]. Pylephlebitis is therefore considered to be a life-threatening illness if not diagnosed and treated before the development of severe sepsis. A cholecystocolonic fistula (CCF), which is one of the cholecystoenteric fistulas, is a rare complication of cholecystitis. Preoperative diagnosis of CCF is difficult, and a misdiagnosis may result in a challenging situation for the surgeon [3]. Herein, we report a rare case of pylephlebitis associated with CCF.

## 2. Case Presentation

**Figure 1:**
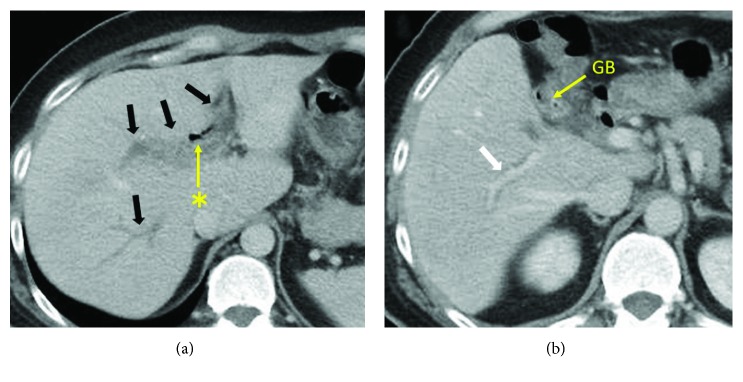
Enhanced computed tomography (CT) showed massive portal vein thrombosis (black arrows). Only the portal branch of segment 6 is patent (white arrow). The gallbladder was collapsed and pneumobilia was seen in the biliary tract (asterisk) (a, b).

**Figure 2:**
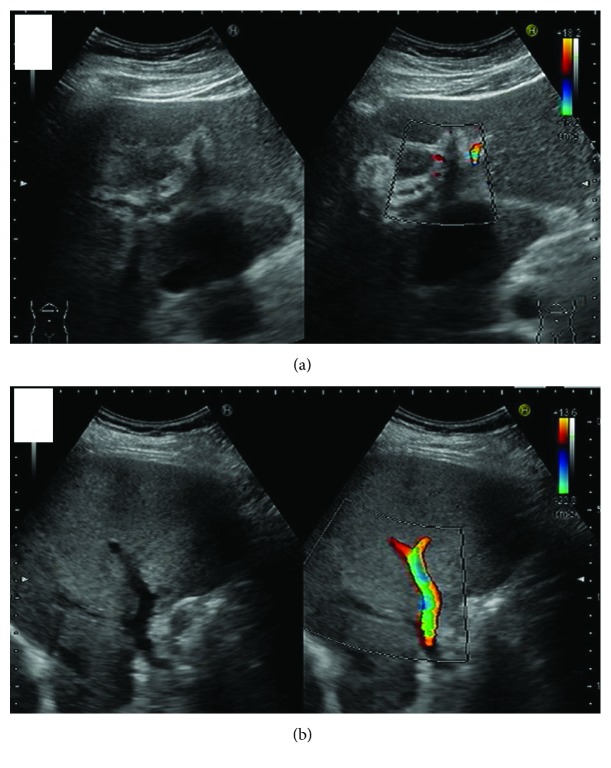
Doppler ultrasonography (US) showed a dilated duct-like structure without any flow in the umbilical portion of the liver (a). Blood flow was detected only in the portal branch of segment 6 (b).

A 41-year-old man was admitted to our hospital due to anorexia, nausea, and constipation. He had experienced severe upper abdominal pain three weeks before admission and the pain had reduced for a few days. His personal history and family history were uneventful. At admission, his vital signs were as follows: temperature, 39.8°C; blood pressure, 147/92 mmHg; and heart rate, 127/min. Laboratory data were as follows: elevated white blood cell count with a left shift, 20650/mm^3^; C-reactive protein (CRP), 14.53 mg/dl; mildly elevated serum levels of aspartate aminotransferase (AST), 70 IU/l; alanine aminotransferase (ALT), 113 IU/l; total bilirubin, 1.5 mg/dl; alkaline phosphatase (ALP), 768 IU/l; and gamma-glutamyl transpeptidase (*γ*GTP), 103 IU/l; prothrombin (PT) activity, 42.3% (PT-INR 1.53); fibrin degradation product (FDP), 149 *μ*g/ml; fibrinolysis degradation product (D-dimer), 1.9 *μ*g/ml; and antithrombin III, 93.1%. Abdominal enhanced computed tomography (CT) showed portal vein thrombosis (PVT) in the left and anterior branch of the portal vein and the wall thickening of the portal vein. The gallbladder was collapsed and pneumobilia was seen in the biliary tract (Figure 1). Doppler ultrasonography (US) revealed dilated duct-like structures without any flow in the liver. Blood flow was detected only in the portal branch of segment 6 of the liver (Figure 2).

**Figure 3:**
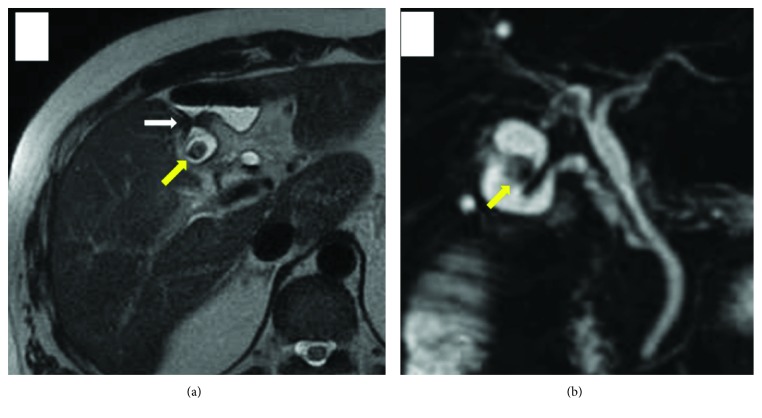
Magnetic resonance imaging (MRI) showed a fistula between the gallbladder and the colon (white arrow). A gallstone in the gallbladder was seen (yellow arrow), but no other gallstone was seen in the common bile duct by cholangiopancreatography (MRCP).

**Figure 4:**
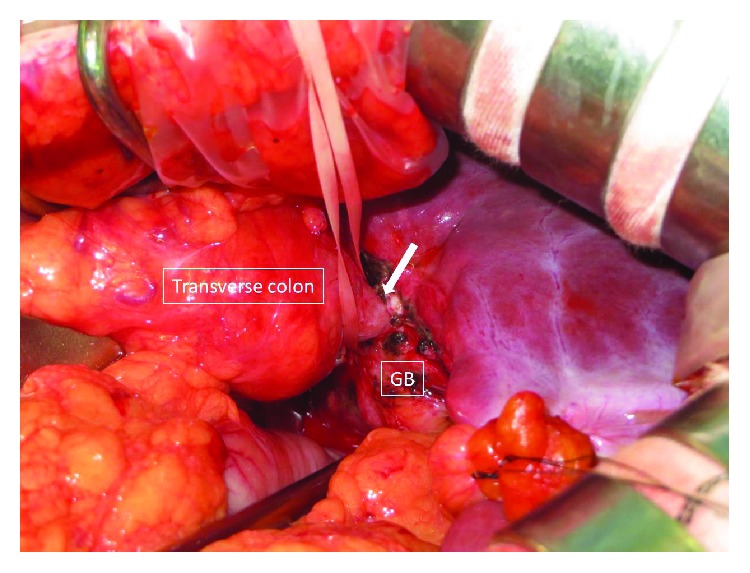
Intraoperative picture indicates that the transverse colon is tightly adherent to the fundus of the gallbladder (white arrow).

A diagnosis of thrombophlebitis of the portal vein associated with CCF was made, and the patient was immediately managed with an intravenous broad-spectrum antibiotic (DRPM at 1.5 g/day) and anticoagulation therapy was started (danaparoid sodium at 2500 IU/day). Blood culture on admission was positive for *Streptococcus anginosus*. Magnetic resonance imaging (MRI) also showed a fistula between the gallbladder and the colon. A gallstone in the common bile duct was not seen by cholangiopancreatography (MRCP) (Figure 3). Follow-up CT scans were performed on hospital days 3 and 13, and it was confirmed that the PVT had not propagated into the main portal vein. On hospital day 13, danaparoid sodium was replaced with heparin (10000 IU/day). The patient's systemic condition gradually improved and laboratory data returned to normal ranges. On hospital day 20, cholecystectomy and partial resection of the transverse colon were performed (Figure 4). Postoperatively, intestinal obstruction occurred, but it was improved conservatively. Heparin was replaced with oral administration of edoxaban tosilate hydrate, and the patient was discharged on postoperative day 50. Although recanalization of the left and anterior branch of the portal vein was not seen, he had no clinical and laboratory abnormalities. Compensatory hypertrophy of the right hepatic lobe and caudate lobe was observed by a CT scan. Administration of edoxaban tosilate hydrate was discontinued at one year after the operation.

## 3. Discussion

Pylephlebitis is defined as a septic thrombophlebitis of the portal vein and its tributaries that is associated with multiple suppurative abdominal infections. This disease was first described by Waller in 1849 as a source of pyogenic intrahepatic abscesses [4]. Due to recent advances in antibiotic therapy, pylephlebitis is now very rare. However, the mortality rate has been reported to be 30%–50% [5, 6]. This high mortality rate is likely to be related to delayed diagnosis because the clinical presentation is frequently nonspecific including fatigue, fever, abdominal pain, nausea, and vomiting [1]. Blood analysis often shows a severe inflammatory reaction, such as leukocytosis and elevated CRP, and mildly abnormal liver function, but these findings are also nonspecific. Death is most commonly due to sepsis or peritonitis, while thrombus propagation into the superior and inferior mesenteric veins may result in bowel ischemia and infarction [7].

The diagnosis of pylephlebitis relies on the demonstration of portal vein thrombosis in the setting of suppurative bacteremia through aspiration of the portal vein. However, portal vein aspiration is seldom performed due to its invasiveness. Instead, surrogate markers such as the patient's clinical condition, imaging, and peripheral blood culture results are used. Among them, CT is the most frequently used diagnostic modality because CT is helpful for making a diagnosis of the exact extent of the thrombus and identifying the source of infection in the abdomen. Bacteremia has been documented in 23% to 88% of patients. The most common bloodstream isolate was *Bacteroides fragilis* followed by *E. coli* and Streptococcus sp. [5].

Once a diagnosis of pylephlebitis is established, appropriate treatment should be started as soon as possible. The treatment of choice is mainly based on broad-spectrum antibiotic therapy. Antibiotic coverage should be targeted towards Gram-positive, Gram-negative, and anaerobic bacteria until culture and sensitivity results are available. The most commonly reported cause of pylephlebitis is diverticulitis, followed by appendicitis, cholecystitis, pancreatitis, and other intra-abdominal infections [1]. To the best of our knowledge, a case of pylephlebitis associated with a cholecystocolonic fistula (CCF) has not been reported in the English literature. CCF is a rare complication of gallstones or gallbladder disease, occurring in 0.06–0.14% of patients with biliary disease [8]. The most common pathogenic mechanism is a chronic inflammatory process of the gallbladder caused by gallstones, resulting in necrosis, perforation, and the development of a fistula [9]. CCF communicates with a bowel lumen with fecal contamination. Therefore, bacteria can flow from the heavily loaded colon to the sterile biliary system and result in various clinical forms of cholangitis including septic shock from biliary sepsis. Barium enema, endoscopic retrograde cholangiopancreatography (ERCP), and colonography have been used in order to demonstrate the fistula in some cases. However, preoperative diagnosis of CCF is difficult and achieved in only 7.9% of patients [3]. Instead, the presence of pneumobilia on abdominal radiographs may provide presumptive evidence for the existence of a biliary-enteric fistula. Other radiological features include a small atrophic gallbladder adherent to neighboring organs or shrunken thick-walled gallbladder around gallstones [10]. In our case, although we did not perform directly certifying procedure such as ERCP, we suspected the existence of CCF from the radiological findings of the pneumobilia and the collapsed gallbladder adherent to the transverse colon on CT and MRI. A definitive diagnosis was achieved during surgical intervention.

The role of anticoagulation therapy in the management of pylephlebitis remains controversial. The aim of anticoagulation is to prevent propagation of the thrombosis and further complications such as portal hypertension and bowel ischemia and to attain portal vein patency. Kanellopoulou et al. analyzed 100 cases of pylephlebitis in the literature and found that patients who received anticoagulation therapy more often had a favorable outcome than did patients who were treated with antibiotics alone [1]. However, the use of anticoagulation is limited to case reports. There has been no study that clearly showed an advantage in terms of improved mortality or clot resolution. Further investigation such as a randomized control study is needed.

Invasive procedures such as surgical thrombectomy can result in rapid recanalization of vessels, but high morbidity and mortality rates have been reported. Percutaneous transhepatic mechanical thrombectomy might also be effective for recent thrombosis, but damage of the vascular wall is frequent and may cause rethrombosis [11]. These invasive treatments are usually not recommended. It is sometimes difficult to determine the timing of surgical treatment for the cause of infection. Early surgical intervention might be considered in cases of appendicitis or in selected patients with acute cholecystitis. However, invasive treatment might result in deterioration of patient's state due to propagation the PVT and development of severe sepsis. Choudhry et al. insisted that treatment should be individualized to the cases [7]. In our case, we strongly suspected CCF preoperatively due to the existence of pneumobilia on CT, and pylephlebitis was thought to be evoked by CCF. At first, improvement of the patient's state is thought to be essential, and intravenous broad-spectrum antibiotic therapy and anticoagulation therapy were immediately started. After the PVT propagation and inflammation had subsided, a delayed operation (cholecystectomy and partial resection of the colon) was performed. The Tokyo Guidelines 2018 presented a management strategy for acute cholangitis and cholecystitis based on an assessment of severity and recommended to perform urgent biliary drainage for severe acute cholangitis [12]. We should have considered early biliary drainage, but the initial treatment including antibiotic and anticoagulant therapy promptly improved the patient's general status. Consequently, we did not perform biliary drainage.

In conclusion, pylephlebitis is a rare but life-threatening complication of intra-abdominal infections if not diagnosed and treated before the development of severe sepsis. Therefore, a high index of suspicion is required and a CT scan should be performed immediately for an early diagnosis and appropriate treatment.
